# Association between overweight, obesity, and quality of life of patients receiving an anticancer treatment for prostate cancer: a systematic literature review

**DOI:** 10.1186/s12955-023-02093-2

**Published:** 2023-01-31

**Authors:** Léonard Depotte, Maryline Caroux, Joseph Gligorov, Florence Canouï-Poitrine, Yazid Belkacemi, Alexandre De La Taille, Christophe Tournigand, Emmanuelle Kempf

**Affiliations:** 1 Assistance Publique – Hôpitaux de Paris, Department of Medical Oncology, Henri Mondor Teaching Hospital, 1 Rue Gustave Eiffel, 94010 Créteil Cedex, France; 2 Department of Medical Oncology, Arras Hospital, Arras, France; 3 Assistance Publique – Hôpitaux de Paris, Department of Medical Oncology, Tenon Teaching Hospital, Paris, France; 4 Assistance Publique – Hôpitaux de Paris, NSERM U955, IMRB-CEpiA Team, Henri Mondor Teaching Hospital, Créteil, France; 5 Assistance Publique – Hôpitaux de Paris, Department of Radiation Therapy, Henri Mondor and Albert Chenevier Teaching Hospital, Créteil, France; 6 Assistance Publique – Hôpitaux de Paris, Department of Urology, Henri Mondor and Albert Chenevier Teaching Hospital, Créteil, France; 7 Sorbonne Université, Inserm, Université Sorbonne Paris Nord, Laboratoire d’Informatique Médicale Et d’Ingénierie Des Connaissances Pour La E-Santé, LIMICS, Paris, France

**Keywords:** Prostatic Neoplasms, Health-related quality of life, Radiotherapy, Prostatectomy, Obesity

## Abstract

**Background:**

Prostate cancer (PCa) and obesity are two ever-increasing public health issues that can independently impair the quality of life (QOL) of affected patients. Our objective was to evaluate the impact of overweight and obesity on the QOL of patients with PCa receiving an anticancer treatment.

**Methods:**

We performed a systematic review of the literature using PubMed, Embase, Cochrane Library and Web of Science databases according to the Preferred Reporting Items for Systematic Reviews and Meta-Analyses. The search equation targeted studies that included PCa patients who had a body mass index (BMI) greater than 25 kg/m^2^, who were receiving anticancer therapy, and whose QOL was analyzed according to validated or non-validated scores.

**Results:**

Of 759 identified articles, we selected 20 studies published between 2000 and 2019 of 12,529 patients treated for PCa, including 5549 overweight or obese patients. QOL assessment was performed using nine validated scales and two non-validated questionnaires. Of seven studies on radiotherapy, six found obesity to have a negative impact on patients' QOL (especially urinary, sexual, and bowel-related QOL). Thirteen studies assessed the QOL of patients who underwent radical prostatectomy, with a BMI > 25 kg/m^2^ having no observed impact. In obese patients under 65 years of age and without comorbidities, nerve-sparing surgery appeared to limit the deterioration of QOL. Four studies on brachytherapy found discordant results. One study showed greater QOL impairment in obese patients receiving first-generation hormone therapy than in those with normal or decreased BMI. No study evaluated the QOL of overweight or obese patients receiving other types of systemic treatment.

**Conclusion:**

Based on the published data, the level of evidence for an association between QOL and overweight or obesity in patients treated for PCa is not high. Prospective cohort studies including this type of patient population are warranted to answer this topical public health issue.

**Supplementary Information:**

The online version contains supplementary material available at 10.1186/s12955-023-02093-2.

## Background

Prostate cancer (PCa) is the second most common form of cancer in men. Nearly 1.5 million were diagnosed with this disease in 2020 [1]. PCa is also the sixth leading cause of cancer death in men, being responsible for 375,000 deaths worldwide in 2020. Depending on stage and aggressiveness as well as on patient age, vulnerabilities, and comorbidities, the treatment options differ. Each is associated with a distinct toxicity profile. Various curative therapies are offered to men with a survival probability of more than 10 years and localized or locally advanced cancer. These are active surveillance, radical prostatectomy, brachytherapy, cryotherapy, and external radiotherapy with or without hormone therapy.

Since the 1980s, quality of life (QOL) has become a major objective in the medical management of oncology patients [2]. In most clinical trials today, it is a key endpoint for treatment approval. Despite recent improvements in radiotherapy and surgical techniques, local treatment of PCa often leads to impaired QOL due to sexual, urinary, and gastrointestinal toxicity. Moreover, anxiety disorders, depression, and fatigue are general adverse effects that can be found regardless of the treatment regimen. Chemical castration by hormone therapy can lead to weight gain or vasomotor symptoms. Because of the relative indolence of PCa, heterogeneity and toxicity of PCa treatments, and clinical condition of patients, QOL is of considerable importance.

In 2018, more than half of American adults reported having a health problem or chronic disease, including obesity [3]. The World Health Organization recognizes obesity as a "global pandemic," with a tripling in worldwide prevalence since 1975. In 2016, nearly 40% of people over 18 were overweight and one in eight adults were obese [4]. Obesity interacts with many chronic diseases, including PCa. Some studies have shown that an elevated body mass index (BMI) is associated with a risk of prostate cancer-specific mortality and biochemical recurrence in PCa patients [5]. Moreover, according to the World Cancer Research Fund, obesity may increase the risk of advanced PCa [6]. However, the influence of obesity on the QOL of patients treated for PCa has only been studied in small sample populations with variable levels of evidence.

The objective is to assess the association between overweight or obesity and QOL in patients who received a treatment for PCa.

## Methods

This systematic review was conducted in accordance with the Preferred Reporting Items for Systematic Reviews and Meta-Analyses statement (PRISMA) [7]. It is registered in the PROSPERO Database of the National Institute for Health Research under number 339197 [8].

We conducted research without any time filter based on PubMed, Embase, Cochrane Library and Web of Science databases. We included any English-language original article that focused on the impact of being overweight or obese on the QOL of patients receiving specific anticancer treatment for PCa. All the definitions of the different items used for the search equations are defined in Additional file 1: Appendix 1. We included studies of patients with PCa regardless of histologic type and tumor stage, except studies on best supportive care only. The treatments were categorized as follows: radical prostatectomy, radiotherapy, or brachytherapy with or without hormone therapy, or systemic therapy alone.

We used the search terms "prostatic neoplasms," "prostate cancer," "quality of life," "overweight," and "obesity". The search equations we used on Pubmed and Embase databases are described in Additional file 1: Appendix 2. Because of the multitude and heterogeneity of QOL assessment scales in PCa, this literature review included all scales to be as exhaustive as possible. No treatment strategies were excluded. Reviews, editorials, and case reports were not included. Eligibility criteria are summarized in Additional file 1: Appendix 3.

Articles were screened by evaluating the title and abstract for the inclusion criteria. Two care providers (MC, EK) reviewed the remaining full text for relevance. Once the final list of studies was obtained, two independent investigators (MC, LD) conducted a double-blind collection of the items of interest according to a previously defined collection grid (Additional file 1: Appendix 4). In the event of a discrepancy, a third independent investigator who is a specialist in medical oncology and methodology (EK) settled the issue. We assessed the risk of bias of the included studies with the NIH Quality Assessment Tool for Observational Cohort and Cross-sectional Studies [9].

## Results

The completed PRISMA 2020 Checklist is shown in Additional file 1: Appendix 5.

### Study selection

**Fig. 1:**
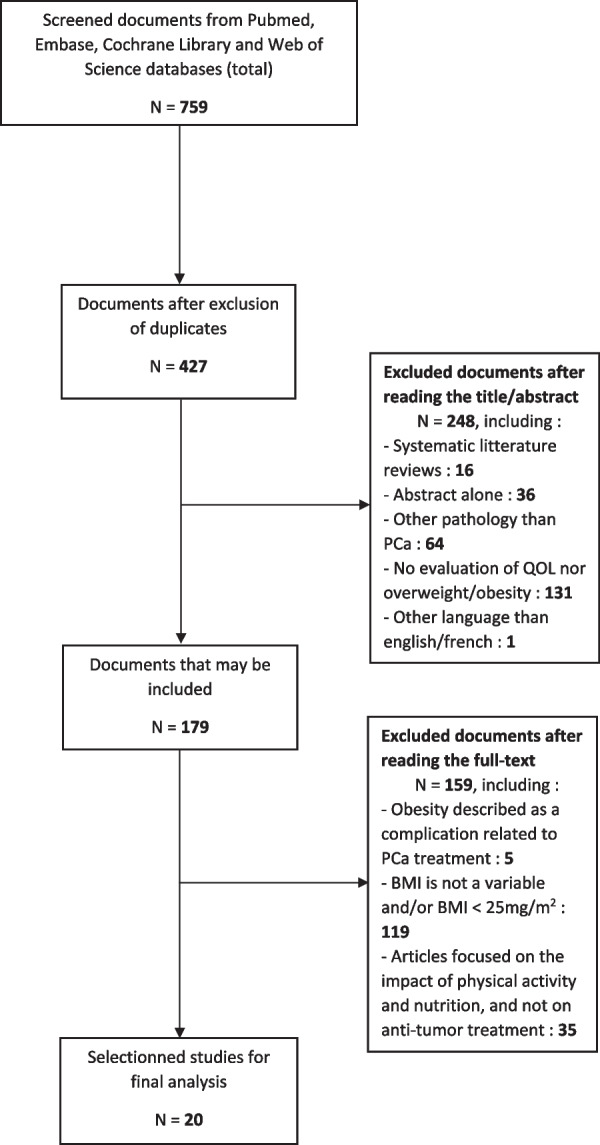
PRISMA study selection flowchart and exclusion criteria

In all, 759 articles were identified from the PubMed, Embase, Cochrane Library and Web of Science databases using the search equation (Fig. 1). After we excluded irrelevant articles, 179 remained. After reviewing the remaining full-text articles for relevance, a total of 20 were identified for inclusion in this review (Table 1). The combined population of those 20 articles was 12,529 patients with PCa, including 5549 overweight or obese patients. These 20 studies were published between 2000 and 2019.

**Table 1:** Main characteristics of the studies included in the systematic review

	References	Objective	Design	Number of patients	Follow-up (months)	QOL scale	QOL evaluation before treatment	BMI categorization (kg/m^2^)	Nerve-sparing status	Patient age	Tumor stage	Comorbidities	Timing and function affected by high BMI
1	Freedland S. [31]	Impact of obesity on QOL after prostatectomy	American, prospective, single-center	340	24	UCLA-PCI	Yes	≤ 25, 25–30, 30–35	Bilateral = 257, unilateral = 68, none = 15	Mean (SD): 56.1 (NA)	T1c-T3a	NA	Urinary function after 24 months
2	Anast J. [11]	Impact of obesity on QOL after prostatectomy	American, retrospective, multicenter	1884	24	UCLA-PCI, SF-36	Yes	≤ 25, 25–30, 30–35, ≥ 35	NA	Mean (SD): 61.5 (NA)	T1-T3	Number of comorbidities (0–1, 2–4)	Physical function, general health, and vitality after 24 months
3	Dess R. [13]	Impact of radiotherapy on erectile function	American, prospective, single-center	373	56	EPIC-26, IIEF-5	Yes	≤ 30, 30–40, ≥ 40	NA	Median (IQR): 69 (64, 73)	T1c-T3	Diabetes, hypertension, coronaropathy, depression	Sexual function after 60 months
4	Latini D. [38]	Impact of diabetes on QOL after prostatectomy	American, prospective, multicenter	1248	24	UCLA-PCI	Yes	≤ 25, 25–30, 30-35	NA	≤ 65: 810, ≥ 65: 438	T1-T3	Number of comorbidities (0, 1–2, 3 +)	Urinary function after 24 months
5	Thomas R. [25]	Impact of obesity on pelvic symptoms post-radiotherapy	English, retrospective, single-center	440	43	CTCAE, Vaizey rectal symptoms score	No	≤ 18.5, 18.5–25, 25–30, ≥ 30	NA	Mean (min, max): 74 (53, 94)	NA	NA	Rectal symptoms and nycturia after 43 months (median)
6	Dieperink K. [20]	Study of QOL after radiotherapy and hormone therapy	Danish, retrospective, single-center	317	Maximum 48 months	SF-12, EPIC-26	No	≤ 30, ≥ 30	NA	Mean (min, max): 65.7 (48, 77)	T1-T3	NA	Urinary symptoms and vitality between 12 and 48 months after treatment
7	Montgomery J. [37]	Impact of obesity on QOL after prostatectomy	American, prospective, single-center	376	36	SF-12, EPIC-26	Yes	≤ 25, 25–30, 30–35, ≥ 35	NA	Mean (SD): 59.2 (NA)	T1-T2	Number of comorbidities (0–1, 2–3, 4 +)	Poorer bowel function after 24 months, if adjuvant radiotherapy
8	Sanda M. [19]	Study of QOL after local treatment (prostatectomy, radiotherapy or brachytherapy)	American, prospective, multicenter	1201	24	EPIC-26, SCA, SCA-P, EPIC-Partner	Yes	Median: 28	Yes	Median (min, max): 63 (38, 84)	T1-T2	Number of comorbidities	Vitality and hormonal function 6 months or later after treatment
9	Wiltz A. [12]	Impact of obesity on QOL after prostatectomy	American, prospective, single-center	945	24	UCLA-PCI, SF-36, IIEF-5, IPSS	Yes	≤ 25, 25–30, 30–35	Yes	Mean (SD): 59.8 (NA)	T1c-T3b	Diabetes, hypertension, coronaropathy	Urinary and sexual function after 12 and 24 months
10	Merrick G. [10]	Impact of obesity on QOL after prostatic brachytherapy	American, retrospective, single-center	32	28	IPSS, IIEF, R-FAS	Yes	≥ 35	NA	Mean (SD): 63.8 (6.5)	T1c-T2b	NA	No difference
11	Ferenc S. [18]	Study of QOL after brachytherapy	Polish, retrospective, single-center	49	2	Numerical scale	No	19–25, ≥ 25	NA	Mean (SD): 66.9 (10.3)	NA	NA	Less urinary incontinence at the end of treatment
12	Alemozaffar M. [15]	Study of sexual function after local treatment (prostatectomy, radiotherapy or brachytherapy)	American, prospective, multicenter	1027	24	EPIC-26	Yes	≤ 25, 25–35, ≥ 35	Yes	Mean (SD): 66 (NA)	T1-T2	NA	Sexual function after 24 months
13	Abdollah F. [14]	Study of urinary incontinence after prostatectomy	Italian, prospective, single-center	1311	12	ICIQ-SF6, IIEF	Yes	≤ 25, ≥ 25	Yes	Mean (min, max): 62.6 (38.9, 80)	≥ T1c	Charlson Index	Urinary incontinence if age < 65, after 3, 6 and 12 months
14	Haahr M. [24]	Study of sexual function after prostatectomy	Danish, retrospective, single-center	704	12	IIEF-5	Yes	≤ 25, 25–30, ≥ 30	Yes	Mean (SD): 62 (5.8)	T1-T3b	Hypertension, diabetes, ASA score, dyslipidemia	Sexual function after 12 months
15	Garg T. [23]	Impact of obesity on sexual and urinary function after prostatectomy	American, retrospective, single-center	691	36	SHIM, IIQ-7, Bladder Health Questionnaire	Yes	≤ 18.5, 18.5–25, 25–30	Yes	Mean (SD): 59.1 (6.4)	T2a-T3b	Diabetes, hypertension, coronaropathy	No difference
16	Limani K. [16]	Impact of BMI on QOL after prostatectomy	Belgian, retrospective, single-center	272	24	ICIQ-UI SF, EORTC QLQ PR25	Yes	≤ 30, ≥ 30	Yes	Mean (SD): 63 (6)	NA	NA	No difference
17	Chen S. [22]	Study of urinary function after local treatment (prostatectomy or radiotherapy)	Taiwanese, prospective, multicenter	131	12	IPSS	Yes	NA	NA	Mean (SD): 68.77 (9.29)	T1-T3	NA	Urinary function after 3 months
18	Cozzi G. [30]	Study of erectile function 1 year after prostatectomy	Italian, retrospective, single-center	643	12	EPIC	Yes	≤ 25, 25–30, ≥ 30	Yes	< 60: 26%, 60–70: 54%, > 70: 20%	T1-T3	Charlson Index	No difference
19	Koneru H. [17]	Impact of obesity on QOL after radiotherapy	American, retrospective, single-center	266	24	EPIC-26	Yes	≤ 25, 25–30, 30–35, 35–40, ≥ 40	NA	Median (min, max): 69 (44, 94)	NA	Charlson Index	Sexual function after 24 months and fatigue after 18 months
20	Challapalli A. [21]	Study of vasomotor symptoms during hormone therapy	English, prospective, single-center	250	9	Vasomotor symptoms scale	No	Median BMI: 28	NA	Mean (SD): 74 (NA)	T1-T4, N0-N1, M0-M1	Hypertension, diabetes, myocardial infarction	Vasomotor symptoms after 9 months

ASA score = American Society of Anesthesiologists; BMI = Body Mass Index; CTCAE = Common Terminology Criteria for Adverse Events; EORTC QLQ-C30 = European Organization for Research and Treatment of Cancer Quality of Life Questionnaire; EPIC = Expanded Prostate Cancer Index; EPIC-Partner = Expanded Prostate Cancer Index for Partners; EQ-5D-5L = 5-Level 5-Dimension EuroQol scale; FACT-P = Functional Assessment of Cancer Therapy – Prostate; ICIQ-UI SF = International Consultation on Incontinence Questionnaire – Urinary Incontinence Short Form; IIQ = Incontinence Impact Questionnaire; NA = Not Applicable; PCa = Prostate Cancer; QOL = Quality of Life; R-FAS = Rectal Function Assessment Score; SCA = Service Satisfaction Scale for Cancer Care; SCA-P = Service Satisfaction Scale for Cancer Care for Partners; SD = Standard Deviation; SF-12 = Short-Form 12

### Study characteristics

The assessment of study bias is summarized in Table 2. The number of patients included per study ranged from 32 [10] to 1884 [11]. None of the studies were randomized, 10 were retrospective, 10 were prospective longitudinal observational studies, 5 were conducted in multiple centers, and 12 were conducted in the last 10 years, including 6 in the last 5 years. None of the studies evaluated castration-resistant metastatic disease, patients on second-generation hormone therapy, chemotherapy, or metabolic radiotherapy. In total, 10 studies highlighted the impact of age on QOL in patients receiving specific anticancer therapy for PCa.

**Table 2:** Bias analysis

	Freedland	Anast	Dess	Latini	Thomas	Dieperink	Montgomery	Sanda	Wiltz A	Merrick	Ferenc	Alemozaffar	Abdollah	Haahr	Garg	Limani	Chen	Cozzi	Koneru	Challapalli
Q1	Y	Y	Y	Y	Y	Y	Y	Y	Y	Y	Y	Y	Y	Y	Y	Y	Y	Y	Y	Y
Q2	Y	Y	Y	Y	Y	Y	Y	Y	Y	Y	Y	Y	Y	Y	Y	Y	Y	Y	Y	Y
Q3	Y	Y	UK	Y	Y	UK	Y	Y	Y	Y	N	UK	N	Y	Y	UK	UK	Y	N	UK
Q4	Y	Y	Y	Y	Y	Y	Y	UK	Y	Y	Y	Y	N	N	N	Y	Y	Y	Y	Y
Q5	Y	Y	Y	Y	Y	Y	Y	Y	Y	N	N	Y	Y	N	Y	Y	Y	Y	Y	N
Q6	Y	Y	Y	Y	N	N	Y	Y	Y	Y	N	Y	Y	Y	N	Y	N	Y	Y	N
Q7	Y	Y	Y	Y	Y	Y	Y	Y	Y	Y	N	Y	Y	Y	Y	Y	Y	Y	Y	Y
Q8	Y	Y	Y	Y	Y	Y	Y	Y	Y	N	N	N	Y	N	Y	N	Y	Y	Y	Y
Q9	Y	Y	Y	Y	Y	Y	Y	Y	Y	N	N	Y	Y	N	Y	Y	N	Y	Y	Y
Q10	Y	Y	Y	N	Y	Y	Y	Y	Y	Y	N	Y	Y	Y	Y	Y	N	Y	Y	Y
Q11	Y	Y	Y	Y	Y	Y	Y	Y	Y	N	N	N	Y	N	Y	N	N	Y	Y	Y
Q12	N	N	N	N	N	N	N	N	N	N	N	N	N	N	N	N	N	N	N	N
Q13	Y	UK	Y	Y	Y	UK	N	N	Y	Y	UK	Y	UK	N	UK	UK	N	Y	UK	UK
Q14	Y	Y	Y	Y	Y	Y	Y	Y	Y	N	Y	Y	Y	Y	Y	Y	Y	Y	UK	N

Y = Yes, N = No, UK = Unknown

1. Was the research question or objective in this paper clearly stated?

2. Was the study population clearly specified and defined?

3. Was the participation rate of eligible people at least 50%?

4. Were all the subjects selected or recruited from the same or similar populations (including the same time period)? Were inclusion and exclusion criteria for being in the study prespecified and applied uniformly to all participants?

5. Was a sample size justification, power description, or variance and effect estimates provided?

6. For the analyses in this paper, were the exposure(s) of interest measured prior to the outcome(s) being measured?

7. Was the timeframe sufficient so that one could reasonably expect to see an association between exposure and outcome if it existed?

8. For exposures that can vary in amount or level, did the study examine different levels of the exposure as related to the outcome (e.g., categories of exposure, or exposure measured as continuous variable)?

9. Were the exposure measures (independent variables) clearly defined, valid, reliable, and implemented consistently across all study participants?

10. Was the exposure(s) assessed more than once over time?

11. Were the outcome measures (dependent variables) clearly defined, valid, reliable, and implemented consistently across all study participants?

12. Were the outcome assessors blinded to the exposure status of participants?

13. Was loss to follow-up after baseline 20% or less?

14. Were key potential confounding variables measured and adjusted statistically for their impact on the relationship between exposure(s) and outcome(s)

### QOL assessment

The choice of scale depended on the purpose of the study. When QOL was assessed in a general way, the SF-36 or RAND 36 questionnaires were used [11, 12]. To evaluate the impact of PCa treatments on one or more functional symptoms affecting urinary, sexual, and gastrointestinal QOL, the authors used validated scales like EPIC 26 (Expanded Prostate Cancer Index, 10 studies), UCLA-PCI (University of California Los Angeles Prostate Cancer Index, 4 studies), EORTC QLQ-PR25 (European Organization for Research and Treatment of Cancer—Quality of Life Prostate, 1 study), IIEF (Index of Erectile Function, 4 studies), SHIM (Sexual Health Inventory for Men, 2 studies), IPSS (International Prostate Symptom Score, 5 studies), ICIQ-SF6 (International Consultation Incontinence Questionnaire Short Form, 2 studies), and Vaizey score for rectal symptomatology [1 study]. Three authors used non-validated questionnaires, and three others aimed to improve QOL assessment in patients treated for PCa by developing new instruments [13–15]. The assessment scales and scores used in the reviewed studies are available in Additional file 1: Appendix 7

### Body mass assessment

All studies focused on BMI divided into categories. Most studies compared normal weight (BMI > 25 kg/m2), overweight (25–30 kg/m2) and obese (> 30 kg/m2), while others only compared obese versus non-obese patients [13, 16, 17] or overweight versus normal weight patients [14, 18]. Three other studies [19–21] only mentioned median BMI and the Taiwanese study [22] did not provide any information on BMI values, categorizing it as either high or normal. Finally, seven studies distinguished between grades of obesity (BMI 30–34.9 kg/m2, 35–40 kg/m2, or greater than 40 kg/m2). This allowed the impact of the grades of obesity to be investigated more precisely.

### Patient characteristics

The median age of the patients included in each study ranged from 59 to 69 years. Patient tumor stages were available in 12 studies, with a mean of 75% of patients having T1 disease in 7 studies. When comorbidities were specifically collected [12, 13, 21, 23, 24], hypertension was the most frequently encountered comorbidity, followed by diabetes and coronary heart disease. Patients' marital status and level of education or standard of living were recorded in seven studies, five of which were American.

### Impact of patient BMI on QOL according to type of anticancer treatment

Only one study [21] on hormone therapy included metastatic patients. Most of the studies involved one treatment or a combination of local treatments. Thirteen articles dealt with prostatectomy, eight with radiotherapy, and five with brachytherapy. Five studies evaluated multiple treatments. None focused on systemic chemotherapy alone.

Due to the heterogeneity of the timing of assessment (3 to 60 months) and of the QOL scores used and the variable definitions of overweight and obesity, it was not possible to perform a quantitative analysis. Among the 13 studies of radical prostatectomy, 5 showed that obesity increased the risk of post-prostatectomy urinary disorders, while 5 others did not find any association. Likewise, 3 studies highlighted a negative relationship between sexual disorders and obesity, while 2 others reported the impact of high BMI on post-prostatectomy vitality. The conclusions of the 6 studies that looked at radiotherapy were more unanimous. Obesity and overweight had a negative impact on QOL, 2 observing an effect on sexual function, 3 on urinary function, and 3 on vitality. These adverse events appeared to occur after a long interval after radiation therapy (12 to 60 months) in 4 of them [13, 17, 20, 25]. Regarding the 4 studies that looked at brachytherapy, 2 found a negative relationship between obesity and QOL on bowel, urinary, and sexual function. Finally, a single 2018 study [21] found increased vasomotor symptoms, fatigue, and insomnia in patients on LHRH analogs who had higher BMI.

## Discussion

This systematic review of 20 studies involving 12,529 patients with PCa, of whom 5549 were overweight or obese, showed that being overweight was more frequently associated with impaired erectile and urinary function, and decreased vitality after radiotherapy. The results after radical prostatectomy and brachytherapy were more discordant, suggesting a possible effect of obesity and overweight on urinary and sexual function. Severe hormone therapy-related toxicity such as vasomotor symptoms occurred more in overweight or obese patients. The impact of high BMI on QOL has never been studied in patients receiving second-generation hormone therapy, chemotherapy, or metabolic radiotherapy with radium-223.

These results may have some explanations. In 2013, a French study suggested that physical, technical, and dosimetric difficulties in radiotherapy may increase acute and late toxicities in patients with higher BMI [26]. In patients undergoing prostatectomy, no study has ever been able to determine the real impact of obesity on QOL. Therefore, some studies recommend prostatectomy in obese patients given the advances in robot-assisted laparoscopic surgery [27]. Conversely, others consider obesity to be a predictor of adverse effects on QOL [28, 29]. In this systematic review, overweight and obesity alone do not seem to impair QOL after prostatectomy.

Several confounding factors may be at work. First, the age of the patients in these 20 studies varied significantly. Aging seems to be associated with a higher risk of post-prostatectomy urinary incontinence [14, 16, 22] and erectile dysfunction [13, 15, 24, 30]. In one study, the impact of obesity on QOL was lower in younger patients [31]. According to the literature, obesity in the elderly and aging tends to favor the appearance of comorbidities and increase complications [32]. But this question remains complex and debated, and some studies seem to show on the contrary better survival outcomes in older, overweight patients treated for cancer [33, 34].

Second, it has long been known that obesity is a risk factor for certain pathologies, such as hypertension, diabetes, or coronary artery disease [35]. These comorbidities and their treatments, independently of weight, can cause erectile dysfunction, reduced libido, and impaired QOL [36]. Comorbidities varied across the 20 studies analyzed in our review. Over half of the obese patients in one of our studies had two to four comorbidities or more [11]. The impairment of physical function, vitality, and global QOL postoperatively was greater in these highly comorbid patients. In contrast, fewer comorbidities appear to correlate with a lower impact of overweight and obesity [23, 30, 37].

Finally, the type of anticancer treatment may have contributed itself to an interpretation bias in some studies. Those that mostly included patients who underwent nerve-sparing surgery concluded that overweight had no impact on postoperative QOL [16, 23, 31]. Other specific patients’ characteristics may explain the choice between radiotherapy and radical prostatectomy for patients with a localized PCa.

To our knowledge, this study is the first literature review to examine the impact of obesity on the QOL of obese patients treated for PCa. This question is crucial from a public health point of view, as these two pathologies are frequent and increasing worldwide. Half of the studies in our literature review were prospective. This allowed us to obtain reliable, although sometimes contradictory, results.

Our literature review has some limitations. None of the studies focused on new systemic therapeutic strategies for PCa, such as second-generation hormone therapy or metabolic radiotherapy. New studies should be carried out to address this little-known issue. The heterogeneity of the patients' characteristics probably affected their QOL, and thus biased our evaluation of its association with overweight and obesity. Finally, the timing of assessment and the scales related to QOL and comorbidities were heterogeneous and may have changed ever since [36].

## Conclusion

The existence of a dual public health issue of PCa and overweight or obesity led us to conduct this literature review, which included 20 studies on impaired QOL in actively treated patients. Our results highlight that overweight and obesity negatively impacted QOL in patients receiving radiotherapy for PCa, particularly their urinary, sexual, and bowel function, 12 months and more after treatment. The results were more discordant in patients receiving prostatectomy or brachytherapy. In this context of rising obesity and PCa, prospective studies evaluating their interaction and the effectiveness of public health measures to combat obesity are expected.

## Supplementary Information

**Additional file 1. Appendix 1**. Items definitions.** Appendix 2**. Search equations.** Appendix 3**. Eligibility criteria.** Appendix 4**. Items of interest.** Appendix 5**. PRISMA 2020 Checklist.** Appendix 6**. Comorbidities Scores.** Appendix 7**. QOL Scales.

## Data Availability

All data analyzed during this study are published articles available online.

## References

[1] Sung H, Ferlay J, Siegel RL, Laversanne M, Soerjomataram I, Jemal A (2021). Global Cancer Statistics 2020: GLOBOCAN estimates of incidence and mortality worldwide for 36 Cancers in 185 countries. CA Cancer J Clin.

[2] Aaronson NK, Ahmedzai S, Bergman B, Bullinger M, Cull A, Duez NJ (1993). The European organization for research and treatment of cancer QLQ-C30: a quality-of-life instrument for use in international clinical trials in oncology. JNCI J Natl Cancer Inst.

[3] Boersma P, Black LI, Ward BW (2020). Prevalence of multiple chronic conditions among US adults, 2018. Prev Chronic Dis.

[4] Obesity and overweight [Internet]. [cited 2022 Jul 10]. https://www.who.int/news-room/fact-sheets/detail/obesity-and-overweight

[5] Cao Y, Ma J (2011). Body Mass index, prostate cancer-specific mortality, and biochemical recurrence: a systematic review and meta-analysis. Cancer Prev Res.

[6] Prostate cancer|What causes prostate cancer?|WCRF International. https://www.wcrf.org/diet-activity-and-cancer/cancer-types/prostate-cancer/

[7] Page MJ, McKenzie JE, Bossuyt PM, Boutron I, Hoffmann TC, Mulrow CD (2021). The PRISMA 2020 statement: an updated guideline for reporting systematic reviews. BMJ.

[8] PROSPERO [Internet]. [cited 2022 Jul 10]. https://www.crd.york.ac.uk/prospero/

[9] Study Quality Assessment Tools | NHLBI, NIH [Internet]. [cited 2022 Sep 26]. Available from: https://www.nhlbi.nih.gov/health-topics/study-quality-assessment-tools

[10] Merrick GS, Butler WM, Wallner K, Galbreath RW, Anderson RL, Kurko BS (2002). Permanent prostate brachytherapy-induced morbidity in patients with grade II and III obesity. Urology..

[11] Anast JW, Sadetsky N, Pasta DJ, Bassett WW, Latini D, DuChane J (2005). The impact of obesity on health related quality of life before and after radical prostatectomy (data from CaPSURE). J Urol..

[12] Wiltz AL, Shikanov S, Eggener SE, Katz MH, Thong AE, Steinberg GD (2009). Robotic radical prostatectomy in overweight and obese patients: oncological and validated-functional outcomes. Urology.

[13] Dess RT, Hartman HE, Aghdam N, Jackson WC, Soni PD, Abugharib AE (2018). Erectile function after stereotactic body radiotherapy for localized prostate cancer. BJU Int.

[14] Abdollah F, Sun M, Suardi N, Gallina A, Tutolo M, Passoni N (2013). A novel tool to assess the risk of urinary incontinence after nerve-sparing radical prostatectomy. BJU Int.

[15] Alemozaffar M, Regan MM, Cooperberg MR, Wei JT, Michalski JM, Sandler HM (2011). Prediction of erectile function following treatment for prostate cancer. JAMA.

[16] Limani K, Albisinni S, Aoun F, le Dinh D, Biaou I, Hawaux E (2017). Qualité de vie après prostatectomie robotique : impact des indices de masse corporelle et âge sur l’incontinence urinaire. Prog Urol.

[17] Koneru H, Cyr R, Feng LR, Bae E, Danner MT, Ayoob M, et al. The impact of obesity on patient reported outcomes following stereotactic body radiation therapy for prostate cancer. Cureus. 2016 Jul 5; 10.7759/cureus.669 PMC4977219 27551649

[18] Ferenc S, Rzymski P, Skowronek J, Karczewski J (2015). Physical and psychosocial side-effects of brachytherapy: a questionnaire survey. J Contemp Brachytherapy.

[19] Sanda MG, Dunn RL, Michalski J, Sandler HM, Northouse L, Hembroff L (2008). Quality of life and satisfaction with outcome among prostate-cancer survivors. N Engl J Med.

[20] Dieperink KB, Hansen S, Wagner L, Johansen C, Andersen KK, Hansen O (2012). Living alone, obesity and smoking: Important factors for quality of life after radiotherapy and androgen deprivation therapy for prostate cancer. Acta Oncol (Madr).

[21] Challapalli A, Edwards SM, Abel P, Mangar SA (2018). Evaluating the prevalence and predictive factors of vasomotor and psychological symptoms in prostate cancer patients receiving hormonal therapy: Results from a single institution experience. Clin Transl Radiat Oncol.

[22] Chen SSS, Cheng TC, Chiu LP, Tasi LY, Huang SS, Tsay SL (2019). Predictors for lower urinary tract symptoms and the urinary specific quality of life in prostate cancer patients: One-year follow-up. J Chin Med Assoc.

[23] Garg T, Young AJ, Kost KA, Park AM, Danella JF, Kirchner HL (2017). Patient-reported quality of life recovery curves after robotic prostatectomy are similar across body mass index categories. Investig Clin Urol.

[24] Haahr MK, Azawi NH, Andersen LG, Carlson S, Lund L (2017). A retrospective study of erectile function and use of erectile aids in prostate cancer patients after radical prostatectomy in Denmark. Sex Med.

[25] Thomas RJ, Holm M, Williams M, Bowman E, Bellamy P, Andreyev J (2013). Lifestyle factors correlate with the risk of late pelvic symptoms after prostatic radiotherapy. Clin Oncol.

[26] Pichon B, Thureau S, Delpon G, Barillot I, Mahé MA (2013). Obésité et irradiation : difficultés techniques, toxicité et efficacité. Cancer/Radiothérapie.

[27] Beyer B, Kühne K, Böhm K, Schiffmann J, Heinzer H, Michl U (2015). Roboterassistierte radikale Prostatektomie. Urologe.

[28] Froehner M, Wirth MP. Re: Vincenzo Ficarra, Giacomo Novara, Raymond C. Rosen, et al. Systematic Review and Meta-analysis of Studies Reporting Urinary Continence Recovery After Robot-assisted Radical Prostatectomy. Eur Urol 2012;62:405–17. Eur Urol. 2013 Mar;63(3):e38. 10.1016/j.eururo.2012.11.037 23201470

[29] Knipper S, Mazzone E, Mistretta FA, Palumbo C, Tian Z, Briganti A (2019). Impact of obesity on perioperative outcomes at robotic-assisted and open radical prostatectomy: results from the national inpatient sample. Urology.

[30] Cozzi G, Musi G, Monturano M, Bagnardi V, Frassoni S, Jereczek-Fossa BA, et al. Sexual function recovery after robot-assisted radical prostatectomy: Outcomes from an Italian referral centre and predicting nomogram. Andrologia. 2019;51(10). 10.1111/and.13385 31423619

[31] Freedland SJ, Haffner MC, Landis PK, Saigal CS, Carter HB (2005). Obesity does not adversely affect health-related quality-of-life outcomes after anatomic retropubic radical prostatectomy. Urology.

[32] Quilliot D, Böhme P, Malgras A, Ziegler O (2013). L’obésité du sujet âgé. Nutrition Clinique et Métabolisme.

[33] Pamoukdjian F, Aparicio T, Canoui-Poitrine F, Duchemann B, Lévy V, Wind P (2019). Obesity survival paradox in cancer patients: Results from the Physical Frailty in older adult cancer patients (PF-EC) study. Clin Nutr..

[34] Martinez-Tapia C, Diot T, Oubaya N, Paillaud E, Poisson J, Gisselbrecht M (2020). The obesity paradox for mid- and long-term mortality in older cancer patients: a prospective multicenter cohort study. Am J Clin Nutr..

[35] Basdevant A (2006). L’obésité : origines et conséquences d’une épidémie. C R Biol.

[36] Droupy S (2005). Épidémiologie et physiopathologie de la dysfonction érectile. Ann Urol (Paris).

[37] Montgomery JS, Gayed BA, Hollenbeck BK, Daignault S, Sanda MG, Montie JE (2006). Obesity adversely affects health related quality of life before and after radical retropubic prostatectomy. J Urol.

[38] Latini DM, Chan JM, Cowan JE, Arredondo SA, Kane CJ, Penson DF (2006). Health-related quality of life for men with prostate cancer and diabetes: a longitudinal analysis from CaPSURE. Urology.

[39] ASA Physical Status Classification System|American Society of Anesthesiologists (ASA). https://www.asahq.org/standards-and-guidelines/asa-physical-status-classification-system

[40] EQ-5D-5L available modes of administration – EQ-5D [Internet]. https://euroqol.org/eq-5d-instruments/eq-5d-5l-available-modes-of-administration/

[41] Karmakar D, Mostafa A, Abdel-Fattah M (2017). A new validated score for detecting patient-reported success on postoperative ICIQ-SF: a novel two-stage analysis from two large RCT cohorts. Int Urogynecol J.

[42] Rosen RC, Riley A, Wagner G, Osterloh IH, Kirkpatrick J, Mishra A (1997). The international index of erectile function (IIEF): a multidimensional scale for assessment of erectile dysfunction. Urology..

[43] Ramanathan R, Mulhall J, Rao S, Leung R, Martinez Salamanca JI, Mandhani A (2007). Predictive correlation between the International Index of Erectile Function (IIEF) and Sexual Health Inventory for Men (SHIM): implications for calculating a derived SHIM for clinical use. J Sex Med..

[44] Almeras C, Zerbib M, Eschwege F, Debré B (2003). ARTICLE ORIGINAL Questionnaire de qualité de vie UCLA/RAND Prostate Cancer Index après radiothérapie externe pour cancer de prostate localisé : retentissement des complications et qualité de vie générale.

[45] Beyer B, Huland H, Feick G, Graefen M (2015). "Expanded prostate cancer index composite" (EPIC-26): Results of functional treatment in patients with localized prostate cancer. Urologe A..

[46] van Andel G, Bottomley A, Fosså SD, Efficace F, Coens C, Guerif S (2008). An international field study of the EORTC QLQ-PR25: a questionnaire for assessing the health-related quality of life of patients with prostate cancer. Eur J Cancer..

